# VoroCrack3d: An annotated semi-synthetic 3d image data set of cracked concrete

**DOI:** 10.1016/j.dib.2024.110474

**Published:** 2024-05-01

**Authors:** Christian Jung, Claudia Redenbach, Katja Schladitz

**Affiliations:** a Rheinland-Pfälzische Technische Universität Kaiserslautern-Landau (RPTU), Gottlieb-Daimler-Straße 48, 67663 Kaiserslautern, Germany; b Fraunhofer-Institut für Techno- und Wirtschaftsmathematik (ITWM), Fraunhofer-Platz 1, 67663 Kaiserslautern, Germany

**Keywords:** Computed tomography, Fracture, Microstructure modeling, Minimum-weight surface, Adaptive dilation, Annotated training data, Image synthesis

## Abstract

Sustainability is an important topic in the field of materials science and civil engineering. In particular, concrete, as a building material, needs to be of high quality to ensure its durability. Damage and failure processes such as cracks in concrete can be evaluated non-destructively by micro-computed tomography. Cracks can be detected in the images, for example via edge-detection filters or machine learning models. To study the goodness, robustness, and generalizability of these methods, annotated 3d image data are of fundamental importance. However, data acquisition and, in particular, its annotation is often tedious and error-prone. To overcome data shortage, realistic data can be synthesized. The data set described in this article addresses the lack of freely available annotated 3d images of cracked concrete. To this end, seven concrete samples without cracks were scanned via micro-computed tomography. Realizations of a dedicated stochastic geometry model are discretized to binary images and morphologically transformed to mimic real crack structures. These are superimposed on the concrete images and simultaneously yield the label images that distinguish crack from non-crack regions. The data set contains 1 344 of such image pairs and includes a large variety of crack structures. The data set may be used for training machine learning models and for objectively testing crack segmentation methods.

Specifications Table

**Table:** 

Subject	Civil and Structural Engineering, Artificial Intelligence, Mathematical Modelling, Computer Vision and Pattern Recognition
Specific subject area	Crack detection and classification in 3d images of concrete using image processing and deep learning Normal concrete (NC), high-performance concrete (HPC), ultra-high-performance concrete (UHPC), air pore concrete; without and with reinforcements (straight steel fibers, crimped steel fibers, hooked-end steel fibers, polypropylene fibers, fibers from glass fiber-reinforced polymer)
Data format	Raw: Concrete: 16-bit 3d grayvalue images (.tif), labels: 8-bit 3d grayvalue images (.tif)
Type of data	3d images (.tif files)
Data collection	The image data were acquired by means of micro-computed tomography (µCT) at Fraunhofer ITWM, Fraunhofer EZRT, and Hochschule Aalen using the CT devices described in Section ‘Experimental Design’. The synthetic crack structures are realizations of a stochastic geometry model which are discretized and morphologically transformed as described in Section ‘Experimental Design’.
Data source location	Fraunhofer-Institut für Techno- und Wirtschaftsmathematik (ITWM) Fraunhofer-Platz 1, 67663 Kaiserslautern, Germany
Data accessibility	Repository name: Zenodo Data identification number: 10.5281/zenodo.10262854 (DOI) Direct URL to data: https://zenodo.org/records/10262854
Related research article	C. Jung, C. Redenbach, Crack modeling via minimum-weight surfaces in 3d Voronoi diagrams, *J. Math. Ind.***13**, 10 (2023). https://doi.org/10.1186/s13362-023-00138-1

## 1 Value of the Data

• 3d image segmentation methods from the field of machine learning such as random forests and convolutional neural networks are usually trained on large annotated data sets. This data set addresses the need of annotated 3d images of cracked concrete. It consists of 1 344 images obtained by superimposing µCT images of concrete with synthetic crack structures and corresponding label images. That is, the ground truths are provided as well.
• The images show seven concrete types, each with four levels of noise. The synthetic crack structures are based on four stochastic geometry models, include several levels of branching, and appear on multiple scales. The variability within this data set makes it unique and useful for several applications.
• First, the data can be used to train machine learning models such as convolutional neural networks to be used for segmenting cracks in 3d images of concrete. Within this context, the goal is to train models that can be used automatically or semi-automatically for segmenting various concrete types and crack structures. Due to its variability, the data set described here can be considered highly suitable for this task.
• Second, based on this data set, crack segmentation methods can be evaluated objectively. It is thus suitable for benchmarking tests, validating robustness, and studying the generalizability of segmentation methods.
• The data set also provides the blank concrete images without cracks as well as the blank label images that contain only the cracks, making it useful for generating additional images that are not necessarily restricted to concrete.

## 2 Background

Annotated data is a valuable foundation in the field of pattern recognition. It is a prerequisite for training or assessing the performance of algorithms for image segmentation.

However, annotated data is often only scarcely available. This holds true especially for 3d images since manually annotating the regions of interest is time-consuming and usually not a trivial task, even for experts. A solution to that problem is offered by the generation of (semi-)synthetic data. Here, the data comes in pairs of input and ground truth images that objectively classify the regions of the input images. This makes them predestined to be used for training machine learning models. Trained on semi-synthetic images, these models were already successfully applied in many contexts such as crack segmentation in concrete [2,[13], [14], [15] and defect segmentation on metal surfaces [12]. Furthermore, segmentation methods – both from classical image processing and machine learning – can be validated objectively [2,3].

In case of 2d images of cracked concrete, several annotated data sets exist [4,5]. Annotated 2d images are also available for the related field of cracks in (road) pavements [9,10]. In a previous study of the authors, 3d concrete images with synthetic cracks as realizations of fractional Brownian surfaces were considered. The annotated data set is freely available [2]. However, it is restricted to cracks on just one scale that appear on just one concrete type.

Here, we consider cracks that are realizations of a more flexible stochastic geometry model – both in terms of crack shape and crack thickness. Cracks are discretized to binary images, morphologically transformed, and embedded into real µCT images of concrete. The images feature high variability regarding concrete backgrounds, noise levels, crack widths and structures. Hence, we consider the data set unique and capable of filling the gap of freely available, annotated 3d image data of cracked concrete. This makes it highly valuable for the image processing, deep learning, materials science, and civil engineering communities.

The crack modeling and discretization procedure was originally proposed in [1]. While the authors of [1] focus on the development of the stochastic modelling approach, this paper describes a new data set based on a variety of CT images of concrete with embedded model realizations.

## 3 Data Description

**Fig. 1:**
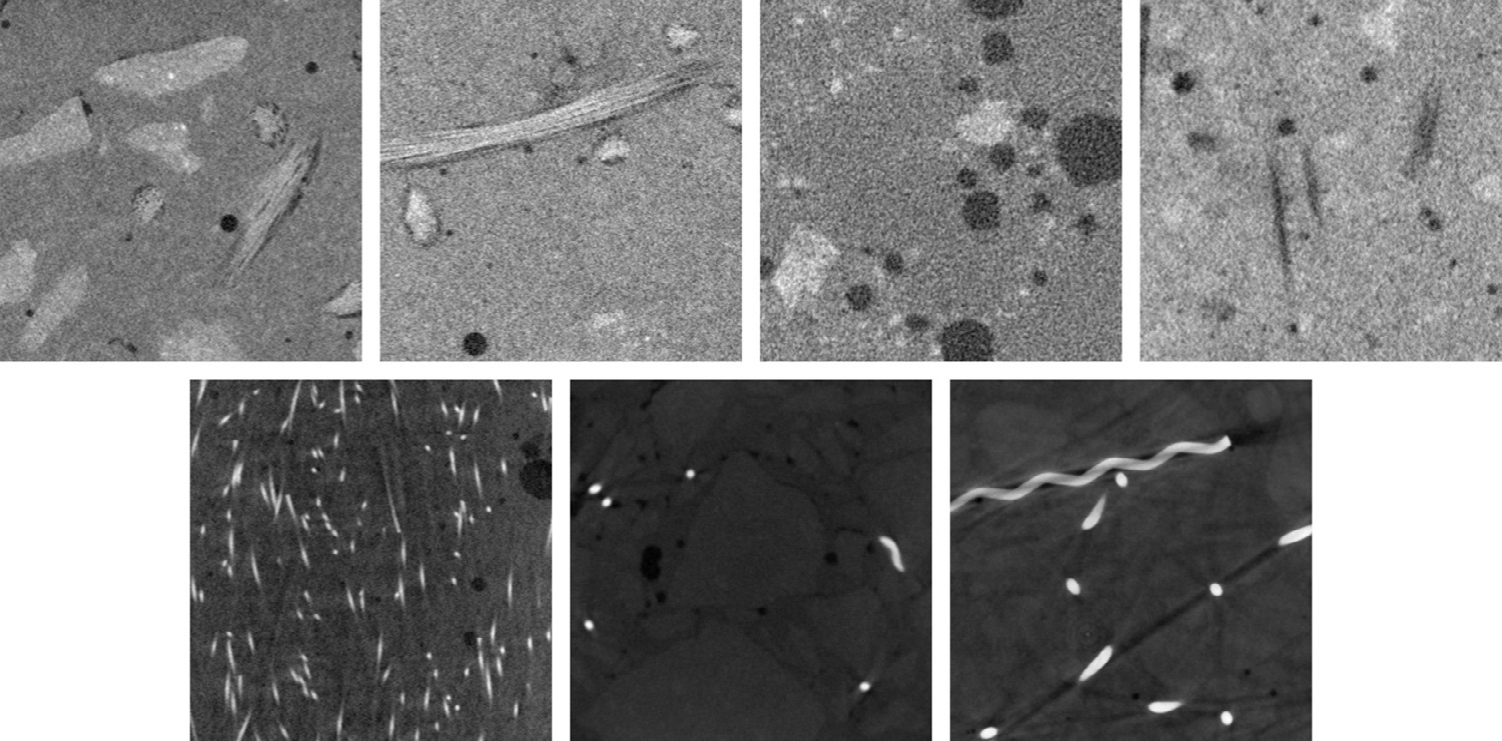
Selected sections of the seven concrete backgrounds. From top left to bottom right: hpc, nc, pores, ppfiber, steelfiber-straight, steelfiber-hooked-end, steelfiber-crimped.

VoroCrack3d [11] comprises a total of 1 344 volume images of concrete. All images are 16-bit 3d grayvalue images in tif-format and of size 400 × 400 × 400 voxels. The data is split up with respect to the seven particular concrete backgrounds shown in Fig. 1. The backgrounds are cropped from reconstructed µCT images of the respective concrete samples. The directory structure is as follows:

1. **hpc**: High-performance concrete reinforced with fibers made of glass fiber-reinforced polymer, voxel size 20.4 µm
2. **nc**: Normal concrete reinforced with fibers made of glass fiber-reinforced polymer, voxel size 22.7 µm
3. **pores**: Air pore concrete, voxel size 2.8 µm
4. **ppfiber**: High-performance concrete, reinforced with polypropylene fibers, voxel size 60.4 µm
5. **steelfiber-crimped**: Ultra-high-performance concrete, reinforced with crimped steel fibers, voxel size 106 µm
6. **steelfiber-hooked-end**: Ultra-high-performance concrete, reinforced with hooked-end steel fibers, voxel size 88.5 µm
7. **steelfiber-straight**: Ultra-high-performance concrete, reinforced with straight steel fibers, voxel size 49.4 µm

For each concrete type, synthetic cracks were generated as a subset of facets of a random Voronoi diagram. We used four different point process models as generators of the diagram to modify the regularity of the cracks. The models are described in the Section ‘Experimental Design’.

The subfolders are split up according to the respective point process models:

A. **hc**: Hard-core point processes (most homogeneous crack shape, moderate local crack shape variation)
B. **matclust**: Matérn cluster point processes (high local shape variation of cracks)
C. **ppp**: Poisson point processes (intermediate local shape variation of cracks)
D. **ppp-scaled**: Poisson point processes; The discretization is scaled by a factor of 2 in the x- and z- direction. (shapes as in C, but with additional anisotropy)

Each of these folders is again split up into five subfolders:

a. **input**: Grayvalue images of concrete with synthetic crack structures
b. **label**: Grayvalue images separating the classes crack (value > 0, corresponding to the local crack width in voxels) and non-crack (value 0)
c. **label-altern**: Same as for ‘label’, except that voxels where cracks intersect pores or steel reinforcements are not considered as crack in the label but have value 0
d. **misc**: Concrete image without cracks, binary image of pore classification via Frangi filter and binary image of steel reinforcement classification via Frangi filter (if applicable)
e. **preview**: 2d preview of one xy-slice (z=200) per 3d input and label image

**Fig. 2:**
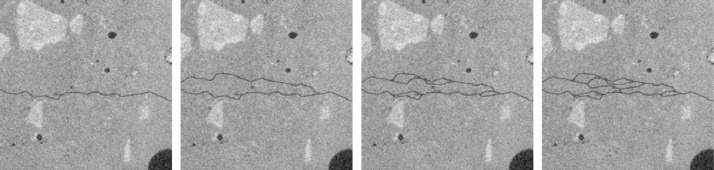
Selected slices from hpc, ppp-scaled, demonstrating several levels of branching. From left to right: 4a, 3a, 2a, 1a.

**Fig. 3:**
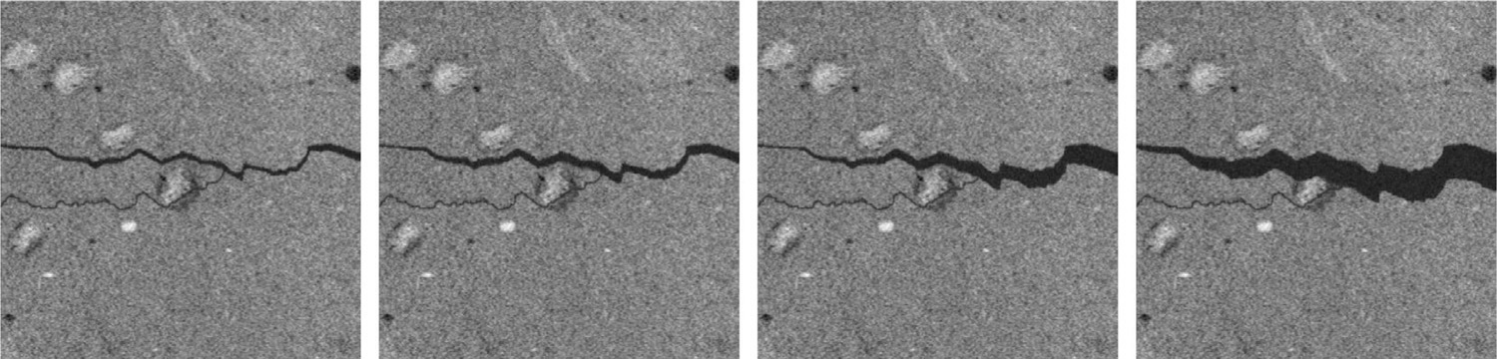
Selected slices from nc, matclust, demonstrating multiscale widths. From left to right: 8a, 9a, 10a, 11a.

The input and label folders then each contain 48 images representing several levels of crack widths and branching (Fig. 2 and Fig. 3). The degree of branching is given by the number of crack branches per image. The local variation of crack width is determined by the Bernoulli parameter, see Section ‘Experimental Design’.

**1a-1d**: crack with up to seven branches; fixed crack width (∼1 voxel)
**2a-2d**: crack with up to four branches; fixed crack width (∼1 voxel)
**3a-3d**: crack with exactly one branch; fixed crack width (∼1 voxel)
**4a-4d**: crack with no branches; fixed crack width (∼1 voxel)
**5a-5d**: crack with no branches; fixed crack width (∼3 voxels)
**6a-6d**: crack with no branches; fixed crack width (∼5 voxels)
**7a-7d**: crack with no branches; fixed crack width (∼7 voxels)
**8a-8d**: crack with up to seven branches; multiscale crack (Bernoulli parameter 0.01)
**9a-9d**: crack with up to seven branches; multiscale crack (Bernoulli parameter 0.02)
**10a-10d**: crack with up to seven branches; multiscale crack (Bernoulli parameter 0.05)
**11a-11d**: crack with up to seven branches; multiscale crack (Bernoulli parameter 0.1)
**12a-12d**: crack with up to seven branches; multiscale crack (Bernoulli parameter 0.2)

**Fig. 4:**
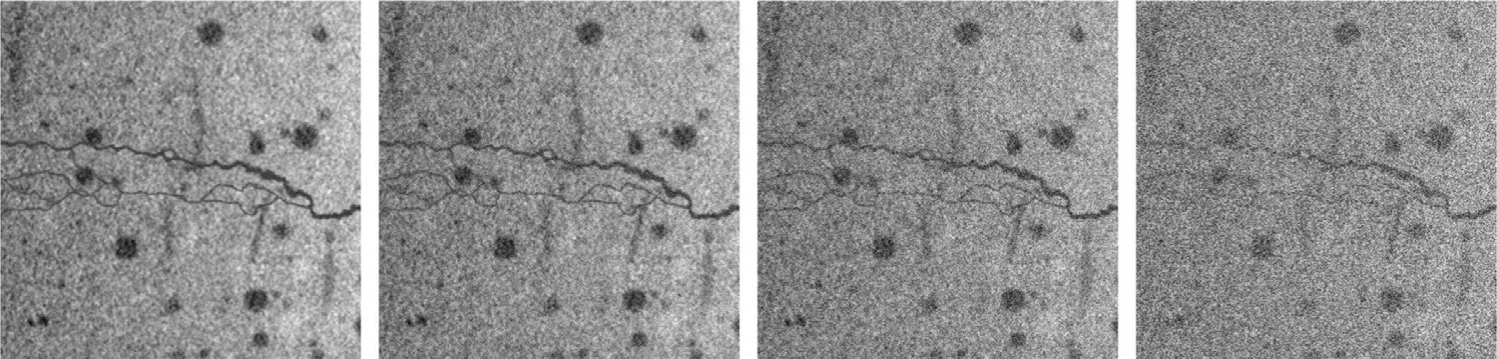
Selected slices from ppfiber, ppp, demonstrating several levels of noise added to the image. From left to right: 8a, 8b, 8c, 8d.

The names ‘**a**’-‘**d**’ indicate the level of noise added to the image. The added values are realizations of independent real-valued random variables drawn from a uniform distribution on an interval given by the grayvalue distribution of the image (with σ being the standard deviation of the grayvalues of the respective concrete background). An example is shown in Fig. 4.

**a**: None
**b**: Noise uniformly distributed on [-σ,σ]
**c**: Noise uniformly distributed on [-2*σ,2*σ]
**d**: Noise uniformly distributed on [-4*σ,4*σ]

The noise values are rounded to the nearest integer and negative values are mapped to 0. Values above $2^{16}$- 1 were not obtained.

## 4 Experimental Design, Materials and Methods

The concrete samples were prepared by Frank Schuler (**steelfiber-hooked-end, steelfiber-crimped**), Bianca Dornisch-Bund (**pores**), Kasem Maryamh (**steelfiber-straight**), Martin Kiesche (**hpc, nc**), and Szymon Grzesiak (**ppfiber**), all at RPTU.

CT imaging was done by Franz Schreiber (**steelfiber-hooked-end, pores, steelfiber-straight, nc, hpc**; at Fraunhofer ITWM), Michael Salamon (**ppfiber**; at Fraunhofer EZRT) and Ralf Löffler (**steelfiber-crimped**; at Hochschule Aalen). The image data was acquired by means of µCT using the hardware setups A-C.

A (ITWM): Feinfocus FXE 225.51 X-ray tube, maximum acceleration voltage 180.3 kV, Perkin Elmer flat-bed detector XRD 1621 with 2 048 × 2 048 pixels.
B (EZRT): Hamamatsu Photonics X-ray tube, maximum acceleration voltage 300 kV, Varex 4 343 HE detector with 3 072 × 3 072 pixels.
C (Hochschule Aalen): phoenix|x-ray Systems X-ray tube, maximum acceleration voltage 240 kV, PaxScan 2 520V detector with 1 920 × 1 536 pixels.

**Fig. 5:**
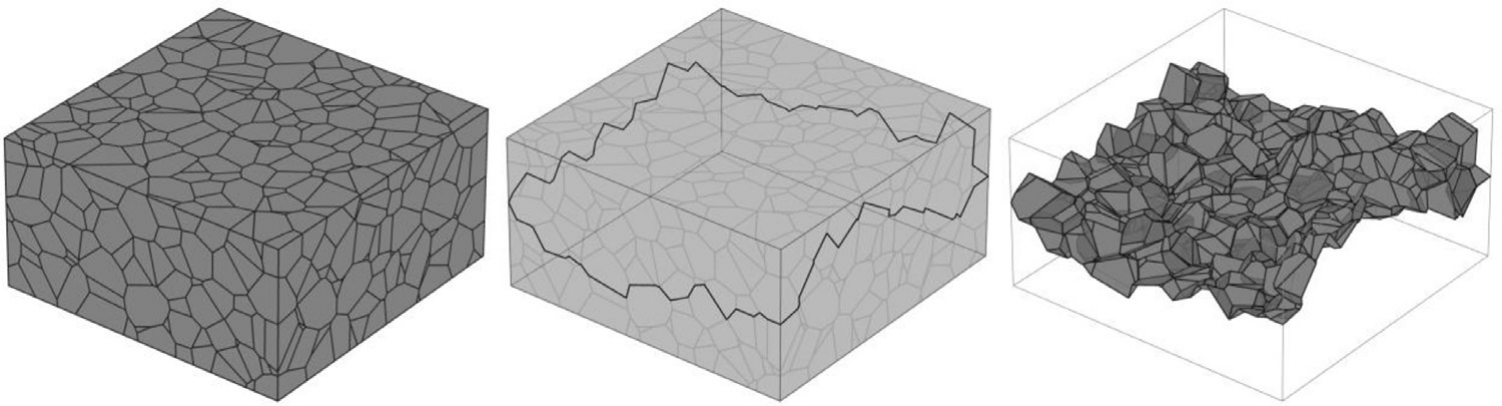
Crack modeling procedure: Voronoi diagram bounded by a cuboid (left), contour on the cuboid that is a set of Voronoi edges (middle), minimum-weight surface inside the Voronoi diagram that is bounded by the contour (right).

The synthetic crack structures are realizations of a stochastic geometry model. In the following, we will present the basic idea behind the modeling procedure and report the parameters that were chosen to produce the cracks. For more details, we refer to [1]. Cracks are modeled via a connected set of facets of a bounded 3d Voronoi diagram. The generators of the Voronoi diagram are realizations of several types of point process models. In the first step, we choose a Voronoi vertex on each of the edges of the bounding cuboid pointing in y-direction. To avoid boundary issues, the vertices are chosen in the center of the cuboid. The four vertices are connected via Dijkstra's algorithm using only Voronoi edges on the faces of the cuboid. The edges are weighted according to their length. This procedure yields a contour on the boundary of the cuboid (Fig. 5, middle). Then, a minimum-weight surface (MWS) bounded by that contour is computed (Fig. 5, right). This is done by solving the linear binary integer program

$$\text{minimize}\sum_{\left\{i:f_{i}\in F\right\}}w\left(f_{i}\right)y_{i}$$

$$\text{subject}\;\text{to}\;Dy=q,y_{i}\in \left\{0,1\right\}$$

where F is the set of Voronoi facets, w a function that assigns each facet a positive weight and D an incidence matrix with $D_{j,i}=1$ if edge $a_{j}$ and face $f_{i}$ are incident and coherent, $D_{j,i}=-1$ if edge $a_{j}$ and face $f_{i}$ are incident and anti-coherent and $D_{j,i}=0$ else. Similarly, for the vector q we have $q_{j}=1$ if $a_{j}$ is part of the contour and coherent to it, $q_{j}=-1$ if $a_{j}$ is part of the contour and anti-coherent to it and $q_{j}=0$ else. After solving the program, $y_{i}=1$ means that facet $f_{i}$ is part of the minimum-weight surface. For more details we refer to [1].

Here, we choose the facet weights to be equal to their respective area.

**Fig. 6:**
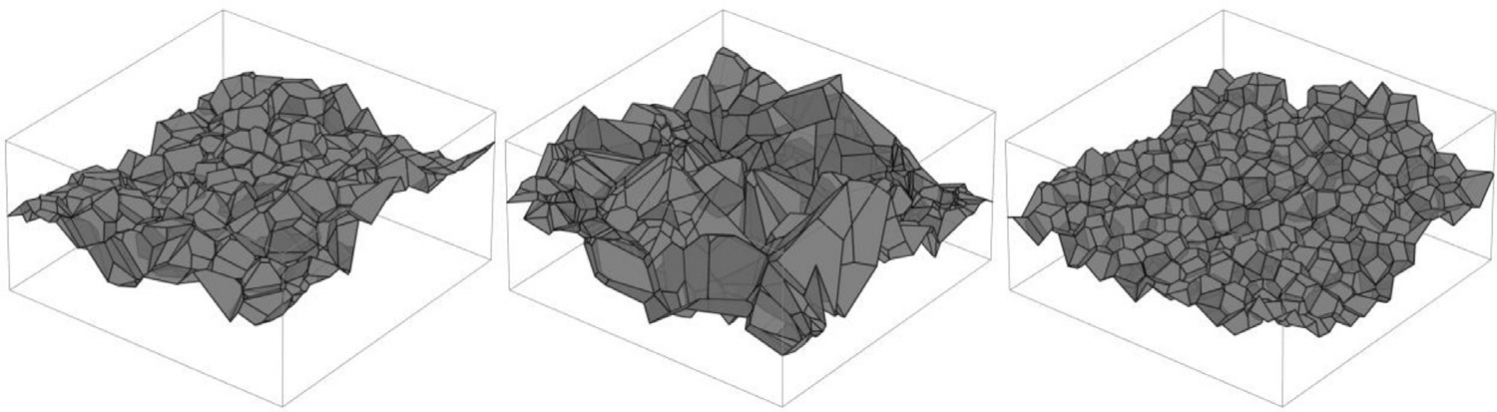
MWS with different levels of regularity. The Voronoi diagarms were generated by a Poisson point process (left), a Matérn cluster process (middle) and a hard-core process (right).

The Voronoi diagram's levels of regularity depends on the chosen underlying point process model. We consider an observation window of size 400 × 150 × 400 and Poisson point processes (‘**ppp**’) with intensity 0.0002, Matérn cluster processes (‘**matclust**’) with parent intensity 0.0002/50, offspring intensity 50, cluster radii 20, and hard-core point processes (‘**hc’**) obtained from force-biased packings of spheres [8] with constant radii, intensity 0.000025 and 60% volume fraction, see Fig. 6. Furthermore, we consider a Poisson point process in an observation window of size 200 × 150 × 200 with intensity 0.0002. The resulting Voronoi diagram will be scaled in the discretization procedure explained below (‘**ppp-scaled**’).

The MWS is then discretized. In the first step, a label image is computed where the grayvalue of each voxel (i,j,k) is set to the index of the Voronoi cell the point (i,j,k) is contained in. In a second step, a binary image is computed by considering adjacent pairs of voxels whose grayvalues in the label image differ. The values of these voxels are set to 1 in the binary image if their labels correspond to cells that generate a facet that is part of the minimum-weight surface. The remaining voxels are set to 0.

In case of the structure in the 200 × 150 × 200 observation window, the discretized image is scaled by a factor of two in x- and z- direction.

Afterwards, the structure is dilated. The cracks with a fixed width are dilated with a structuring element of fixed size (3 × 3, 5 × 5, 7 × 7) or not dilated at all. For the multi-scale cracks, we choose an adaptive dilation procedure: Starting from slice x=1, every 2d slice is dilated separately and repeatedly with a 2 × 2 structuring element. The number of repetitions is derived from a random walk with Bernoulli-distributed increments with parameter p (Bernoulli parameter). We chose p from {0.01, 0.02, 0.05, 0.1, 0.2}.

To model the rough crack boundary, a second Voronoi diagram is generated from a Poisson process in 400 × 150 × 400 with intensity 0.2. This diagram is also discretized. The expected size of a cell of this diagram is roughly 5 voxels. Every cell of this second diagram that touches the initial dilated crack is merged to the crack. For cracks which have not been dilated, this procedure yields widths which exceed the desired width of 1 voxel. In this case we only consider crack voxels that are adjacent to the lower part of the background to be part of the crack.

Crack branching is realized by combining several cracks. The main crack is generated as described above. Branches are added as follows: For the contour, we take two of the vertices that were used for the contour of the main crack. The other two are chosen equally randomly from the respective edges of the cube. Branches are not dilated. For modeling the rough boundary, the same Voronoi diagram as above is used.

**Fig. 7:**
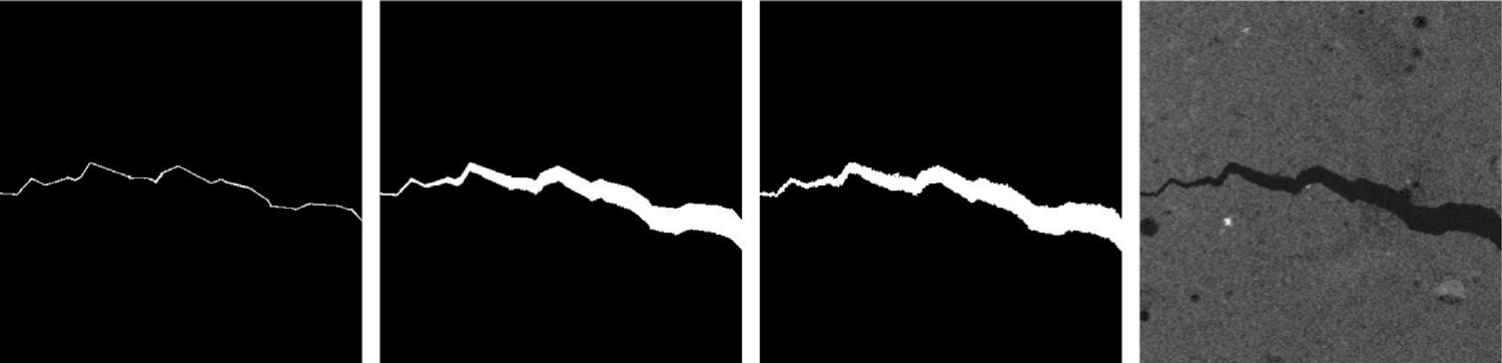
From left to right: Discretization of the MWS, adaptively dilated crack, crack boundary refinement, crack embedded into a CT image of real concrete.

The resulting label image is then padded to a size of 400 × 400 × 400 to obtain the ground truth image. It is embedded into the 3d image of uncracked concrete: The crack's grayvalues are sampled from a normal distribution. Mean and variance are estimated from the empirical distribution of the concrete's air pores. To this end, the air pores are segmented via a Frangi filter [6] for dark blob-like structures on a brighter background. Finally, a Gaussian filter with σ=0.6 is applied to the transition area of crack and concrete background to mimic the partial volume effect. The dilation and embedding procedure is shown in Fig. 7.

**Fig. 8:**
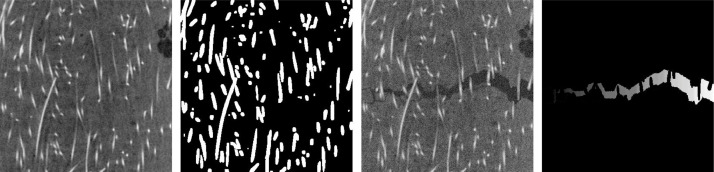
From left to right: Steel fiber-reinforced concrete, steel fiber mask, embedded crack, alternative ground truth.

Steel reinforcements are not expected to fail during crack propagation. Hence, the synthetic cracks do not go through these reinforcements. That is, the fiber system from the background image should be unaffected by the crack embedding described above. To this end, we compute a mask by segmenting the steel reinforcements via a Frangi filter for bright tube-like structures on a darker background. An example is shown in Fig. 8. Cracks are then only added outside the mask.

These considerations also yield the conclusion that an alternative ground truth is beneficial. First, the data set includes a ground truth of the whole crack structure (‘label’). For the second (alternative) ground truth (‘label-altern’), the intersections of crack and fibers are computed. These intersections are then considered background (no crack, value 0) in the ground truth since we assume only uncracked fibers (Fig. 8, right).

**Fig. 9:**
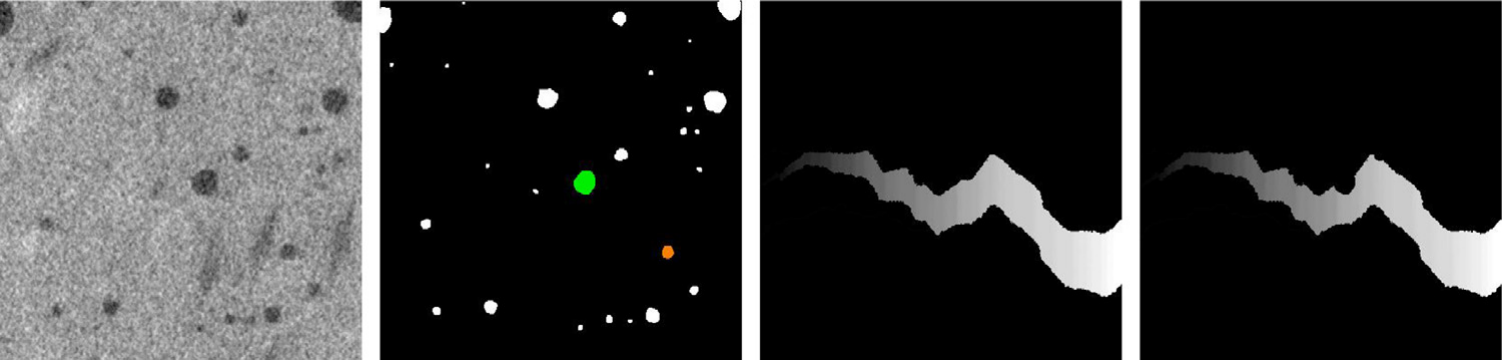
From left to right: Polypropylene fiber-reinforced concrete, air pore segmentation, label and alternative label image. The green pore in the segmentation intersects the crack but is not fully contained in it. Thus, it is not considered part of the crack and is given value 0 in the alternative label. The orange pore is fully contained in the crack. Thus, it is part of the crack and is not considered background.

Also, it can be argued whether air pores intersecting a crack should be considered part of the crack. Here, the intersection of a crack and an air pore is considered background (no crack) if the crack does not contain the whole air pore. The concept is visualized in Fig. 9.

The label of the ground truth grayscale images corresponds to the local crack thickness. The images can be thresholded with threshold 1 to obtain binary label images.

## Limitations

Our crack modeling procedure is based on the assumption that cracks are connected structures which possibly exhibit multiple levels of thickness. Typically, these kinds of cracks appear in concrete samples that were exposed to stress tests such as tensile or bending tests. However, other crack structures are possible. For example, cracks that emerge from alkali-silica reactions typically exhibit a different topology [7]. In particular, a network of single, disconnected cracks can be observed. Our model does not account for these kinds of structures, such that the data set is limited to connected crack structures.

## Ethics Statement

The authors have read and followed the Data in Brief's ethical requirements. We confirm that this work does not involve human subjects, animal experiments, or any data collected from social media platforms.

## CRediT authorship contribution statement

**Christian Jung:** Conceptualization, Methodology, Software, Validation, Data curation, Writing – original draft, Visualization. **Claudia Redenbach:** Validation, Resources, Data curation, Writing – review & editing, Supervision, Funding acquisition. **Katja Schladitz:** Validation, Resources, Data curation, Writing – review & editing, Supervision, Funding acquisition.

## Data Availability

VoroCrack3d (Original data) (Zenodo)
